# Motion-Encoded Electric Charged Particles Optimization for Moving Target Search Using Unmanned Aerial Vehicles

**DOI:** 10.3390/s21196568

**Published:** 2021-09-30

**Authors:** Mohammed A. Alanezi, Houssem R. E. H. Bouchekara, Mohammad S. Shahriar, Yusuf A. Sha’aban, Muhammad S. Javaid, Mohammed Khodja

**Affiliations:** 1 Department of Computer Science and Engineering Technology, University of Hafr Al Batin, Hafr Al Batin 31991, Saudi Arabia; alanezi.mohd@uhb.edu.sa; 2 Department of Electrical Engineering, University of Hafr Al Batin, Hafr Al Batin 31991, Saudi Arabia; mshoaib@uhb.edu.sa (M.S.S.); shaaban@uhb.edu.sa (Y.A.S.); 3 Department of Electrical and Electronic Engineering, Faculty of Engineering, Imperial College London, London SW7 2AZ, UK; mj419@ic.ac.uk; 4 Department of Electronics, College of Engineering, Mustaqbal University, Buraidah 51452, Saudi Arabia; medkhodja28@gmail.com; 5 Department of Electrical Engineering, Faculty of Technology, M’sila University, M’sila 28000, Algeria

**Keywords:** electric charged particles optimization, motion-encoded, probabilistic target finding, unmanned aerial vehicles

## Abstract

In this paper, a new optimization algorithm called motion-encoded electric charged particles optimization (ECPO-ME) is developed to find moving targets using unmanned aerial vehicles (UAV). The algorithm is based on the combination of the ECPO (i.e., the base algorithm) with the ME mechanism. This study is directly applicable to a real-world scenario, for instance the movement of a misplaced animal can be detected and subsequently its location can be transmitted to its caretaker. Using Bayesian theory, finding the location of a moving target is formulated as an optimization problem wherein the objective function is to maximize the probability of detecting the target. In the proposed ECPO-ME algorithm, the search trajectory is encoded as a series of UAV motion paths. These paths evolve in each iteration of the ECPO-ME algorithm. The performance of the algorithm is tested for six different scenarios with different characteristics. A statistical analysis is carried out to compare the results obtained from ECPO-ME with other well-known metaheuristics, widely used for benchmarking studies. The results found show that the ECPO-ME has great potential in finding moving targets, since it outperforms the base algorithm (i.e., ECPO) by as much as 2.16%, 5.26%, 7.17%, 14.72%, 0.79% and 3.38% for the investigated scenarios, respectively.

## 1. Introduction

Currently, unmanned aerial vehicles (UAVs) are among the most promising research tools of interest due to their potentials for use in numerous practical applications [1,2,3]. UAVs are especially suitable for surveillance and rescue tasks. They are highly capable of working under harsh environmental conditions. Moreover, they can connect with a sensor-rich work environment, ideal for dealing with a variety of tasks [4]. In the case of searching for a lost target using UAVs, several factors are taken into consideration. One of those is the ‘golden time’, a critical period when the probability of finding the target becomes maximal [5]. This probability decreases rapidly with time, due to several factors such as the terrain, weather conditions, attenuation of the initial particulars, the target dynamics, etc.

Therefore, while formulating the problem of searching for a lost target by UAVs, it is required to find a path that can maximize the target detection probability within a given flight time. Usually, the target position information and other search conditions play crucial roles in such formulations [6,7]. Such search problems are found in the literature as probability functions where uncertainties are considered using initial assumptions. Besides, sensor models and search conditions are adequately incorporated in the problem formulation. Articles [6,8] proposed a Bayesian approach to deal with the objective functions of detection probability evaluation of UAV flight paths.

Article [9] proposed a novel path tracking mechanism for high-speed autonomous vehicles based on the combination of model predictive control (MPC) and a PID-speed controller. A lane-level multilayer map model has been proposed in [10] to ease the tracking of a UAV or any other autonomous vehicle, even in a complicated network. A self-assessment based method [11] was used to solve a cooperative search problem, where autonomous vehicles can cooperate. This method was checked on different communication structures and excellently performed in scalability, design complexity, and communication skill. The UAV-based search problem is represented as a control system problem in [12] where the detection and collection of data can work simultaneously with coverage control.

In [7,13], the stochastic Markov process was proposed to represent deterministic target dynamics, meaning that the search scenarios will not affect the process. The initial search map can be modeled as a multivariate normal distribution, where the mean and the variance are computed using the initial information of the target position. While modeling the sensor, continuous Gaussian variable [6] and binary variable [14] are both used with the binary states defined as ‘‘detected’’ and ‘‘non-detected’’.

Because of the involvement of multiple probabilistic variables, such search problems are complex in nature.

The complexity of the search problem varies between NP-hard [15] to NEXP-complete [16]. The former refers to the nondeterministic polynomial-time hardness, while the latter refers to nondeterministic exponential-time completeness. In NEXP-complete type problems, the number of solutions increases exponentially along with the flight time and search dimension. Therefore, solving such a problem by classical methods like differential calculus to find the exact solution is understandably impractical. Therefore, approximated heuristic methods are mostly used. Typical optimization algorithms used in this field include genetic algorithms (GA) [17], ant colony optimization (ACO) [14], one-step look ahead greedy search algorithm [6], k-step look ahead greedy search algorithm [7], Bayesian optimization approaches (BOA) [8], branch and bound approach [18], cross-entropy optimization (CEO) [19], gradient descend methods [20,21], limited depth search algorithms [22], neural networks [23] etc. Among the used algorithms, Refs. [8,14,19,22] presented are the algorithms capable of using more than one UAV for search purposes, thus speeding up the search process.

On the other hand, Refs. [6,7] proposed algorithms to support a specific design of the search problem. However, it is noted that most of the used methods can successfully track moving targets using the binary model of sensor detection. Recently, [24] proposed using motion-encoded particle swarm optimization (MPSO) to solve UAVs’ moving-target search problem. The paper also compared the results with some well-known metaheuristic approaches. It was 24% more efficient in detection and 4.71 times more time-efficient than the original PSO. A review paper [25] nicely gathers all the intelligent optimization algorithms that have been tried on UAV dependent swarm search applications.

The literature identifies different approaches adopted to certify the optimal search divergence, such as target dynamics, constraints, assumptions, and searching mechanisms. The complex nature of optimal search, particularly for fast-moving targets, makes optimal problem formulation and solution strategizing challenging. However, recent advancements in sensors, UAV technologies, and communications have opened up new opportunities for research in the field. The solution must be highly robust in terms of search capacity and possess the properties of a methodical optimization tool like adaptability, computational efficiency, optimality, etc.

Electric charged particle optimization (ECPO) is a new metaheuristic optimization algorithm that was proposed by Bouchekara in 2020 [26]. The algorithm is established based on the interaction phenomenon between electrically charged particles. Different strategies of interacting behavior have made this algorithm a robust one and suitable for application on diverse applications. ECPO is a promising optimization tool that has been compared with several state-of-the-art optimization algorithms. It was tested and verified with a set of 30 functions and one real-life engineering problem. This algorithm resembles an intuitive sense of the motion of an apparently stray and lost animal in the wilderness and thus is expected to give results of analogical significance. However, motion encoding feature has been added with the ECPO in this paper for the first time to improve the searching capabilities of the algorithm for UAV dynamic target search problems.

This paper proposes a motion-encoded electric charged particle optimization (ECPO-ME) algorithm to solve the problem of moving target search. Some of the main features of the paper are highlighted below:

- The formulation of the optimization problem with a suitable objective function and the required constraints represents the targeted problem accurately.
- The use of motion-encoding mechanism with ECPO to increase the efficacy of the algorithm. This duo has neither been tried before in the literature nor in solving any optimization problems.
- Comparing the proposed mechanism with 10 commonly used metaheuristic optimization algorithms strengthens the logic of using it in moving target search applications. It is also compared with MPSO, used in a recently published research paper to solve a similar optimization problem.
- The presentation of the convergence curves for all the used optimization methods in a single plot to ease the comparison of their performance.

The simulation results demonstrate the superiority of ECPO-ME over all other optimization algorithms in solving the problem of moving target search, which is crucial in ascertaining the presence and position of a misplaced animal in vast landscapes. Additionally, we prove that the motion-encoding strategy improved the efficacy of the ECPO algorithm by a considerable margin.

The remaining paper is structured as follows: Section 2 contains the problem formulation and the objective function development. Section 3 presents the optimization algorithm details to solve the addressed problem. Section 4 exhibits the simulation problem, and, finally, Section 5 draws the concluding remarks of the article.

## 2. Problem Formulation

The problem addressed in this work is formulated as a search problem that entails modeling of the target, sensor, and belief map, as presented in [24]. These three aspects of the problem formulation are discussed in Section 2.1, Section 2.2 and Section 2.3 respectively and the objective function is developed in Section 2.4.

### 2.1. Target Model

Consider the variable x∈Χ, which represents the unknown location of the target in the search problem. To begin the search, the probability distribution function (PDF) which could be any suitable probability distribution based on the target’s most recent information (for example the target’s most recently known position) is used to model the target’s location. For example, reference [24] discussed using a normal distribution centered on the last known location or a uniform distribution if there is no information about the target’s location. The PDF is represented using the belief map, $b\left(x_{0}\right)$, which is a grid map with each cell marked with the probability of the target being in that cell. The belief map is developed by discretizing the search space into a grid of $S_{r}\times S_{c}$ cells with their associated probabilities such that if the target is in the search space, then $\sum_{\left(x_{0}\in S\right)}b\left(x_{0}\right)=1$.

The target navigation pattern can be modelled as a Markov process. In this work, we assume a conditionally deterministic target whose navigation pattern depends upon the target’s initial position,$x_{0}$. Therefore, probability that a target moves from cell $x_{t-1}$ to $x_{t}$, $p\left(x_{t}|x_{t-1}\right)$ called the transition function is known for all cells $x_{t}\in S$. As such, if the target’s initial position is known, then the target’s entire path will be known, which is a standard assumption in target search problems [14].

### 2.2. Sensor Model

The UAV is equipped with a sensor that carries out independent observations $z_{t}$ at each time step t. An observation is classified based on the results of a detection algorithm; if detected, $z_{t}=D_{t}$ otherwise, $z_{t}={\bar{D}}_{t}$. However, the observation likelihood, $p(z_{t}|\chi_{t})$, given sensor model, captures the fact that $z_{t}=D_{t}$ does not guarantee the presence of a target, due to imperfection of the sensor and detection algorithm. Hence it is quite clear that, for a target location $\chi_{t}$, the likelihood of no detection can be obtained as:

(1) $$p\left({\bar{D}}_{t}|\chi_{t}\right)=1-p(D_{t}|\chi_{t})$$

Here$\;p(D_{t}|x_{t}{}_{t})$ and $p\left({\bar{D}}_{t}{|x}_{t}\right)$ represent the likelihood of detection and the likelihood of no detection, respectively.

### 2.3. Belief Map Update

Given sensor observations, $Z_{1:t}=\left\{z_{1},\;\ldots ,\;z_{t}\right\}$ and initial distribution $b\left(\chi_{0}\right),$ a Bayesian approach can be used to develop the belief map of the target, $b\left(\chi_{t}\right)$. As with all Bayesian approaches, prediction and update operations are required. With the help of Bayesian approaches, both the prediction and update operations can be carried out. For the prediction step, the target motion model is used to propagate the belief map over time according to the equation:

(2) $$\hat{b}\left(x_{t}\right)=\sum_{x_{t-1}\in S}p\left(\chi_{t}|\chi_{t-1}\right)b\left(\chi_{t-1}\right)\;$$

where: $b\left(\chi_{t-1}\right)$ is the previous belief map such that $b\left(\chi_{t-1}\right)=p\left(\chi_{t-1}|z_{1:t-1}\right)$ i.e., the conditional proability of target being at $\chi_{t-1}$ given observations up to t−1; and the update step involves multiplying the latest conditional observation by $\hat{b}\left(\chi_{t}\right):$

(3) $$b\left(x_{t}\right)=\eta p\left(z_{t}|\chi_{t}\right)\hat{b}\left(x_{t}\right)$$

The normalization factor $\eta_{t},$ which measures the probability that the target is inside the search area (i.e., $\sum_{\chi_{t}\in S}b\left(\chi_{t}\right)=1$), is defined as:

(4) $$\eta_{t}=1/\sum_{\chi_{t}\in S}p(z_{t}|\chi \_t)\hat{b}\left(x_{t}\right)$$

### 2.4. Objective Function

Bayesian theory considerations mean that the probability that the target is not detected at time t during any observation, $r_{t}=p({\bar{D}}_{t}|z_{1:t-1})$ depends upon two factors: the latest belief map from the prediction phase (2), and the no-detection likelihood (1). This probability $r_{t}$ is defined as:

(5) $$r_{t}=p\left({\bar{D}}_{t}|\chi_{t}\right)\hat{b}\left(x_{\chi_{t}}\right),$$

which is applicable across the entire search space. From (4) and (5) $r_{t}=1/\eta_{t},$ and $z_{t}={\bar{D}}_{t}$ represents a “no detection” event. The combined probability of failing to detect the target over the period of 1 to t $R_{t}=p\left({\bar{D}}_{1:t}\right)$ becomes:

(6) $$R_{t}=\prod_{k=1}^{t}r_{k}=R_{t-1}r_{t}$$

And the probability of detecting the target for the first time at time t is given as:

(7) $$p_{t}=\prod_{k=1}^{t-1}r_{k}\left(1-r_{t}\right)=R_{t-1}\left(1-r_{t}\right)$$

Therefore, the probability of detecting the target in t steps is a summation of $p_{t}$ over t steps known as the cumulative probability $P_{t}$ is given by:

(8) $$P_{t}=\sum_{k=1}^{t}p_{k}=p_{t-1}+p_{t}$$

Note that the cumulative probability $P_{t}=1-R_{t}$ is different from $p_{t}.$ Hence for a finite search time over a time period $\left\{1,\;\ldots ,\;N\right\},\;$the goal of the search strategy is to determine a search path $O=\left(o_{1},\;\ldots ,\;o_{N}\right)$ that could maximize the cumulative probability $P_{t}$. Therefore, the search objective function is:

(9) $$J=\sum_{t=1}^{N}p_{t}$$

## 3. Motion-Encoded Electric Charged Particles Optimization (ECPO-ME) Algorithm

### 3.1. Description

The ECPO [26], being inspired by the electric charged particles (ECPs) interactions, is a population-based heuristic algorithm. In the description of the algorithm, the following internal parameters are used:

nECP: the total number of ECPs,
MaxITER: the maximum number of iterations,
nECPI: the number of ECPs which are interacting with themselves in one of the three strategies,
naECP: the archive pool size.

The ECP is assumed to search for a better solution by consequent interaction of charged particles in the selected strategy. While interacting, the best particle attracts the comparatively worst one, whereas the worst particle repels the best one. The ECPO is explained in detail in the following sub-section.

### 3.2. Pseudocode

Algorithm 1 presents the pseudocode for ECPO. All the steps are elaborately described in the following sub-sections.
**Algorithm 1** ECPO pseudocode1 **Inputs**ObjFunction (objective function), ProblemSize (dimension of the problem), nECP (number of ECPs), nECPI (number of ECPs in interaction), Strategy, naECP (size of the archive pool) and MaxITER (maximum number of iterations)2**Output**${\mathrm{ECP}}_{\mathrm{best}}$3**Initialization()**4**for** Iter = 1: MaxITER5    **Selection()**6    **Interaction()**7    **BoundsCheck()**8    **Diversification()**9       **PopulationUpdate()**10**end for**

### 3.3. Algorithm

#### 3.3.1. Initialization

The working procedure of ECPO starts by generating nECP charged particles within the search space, like all other metaheuristic approaches. The particles are then sorted according to their fitness. To generate the charged particles, a random normal distribution procedure has been adopted.

#### 3.3.2. Archive Pool

Along with the generated population, a separate archive of naECP is created with the best ECPs and is denoted by archiveECP. In this archive, only the best quality ECPs are stored and updated with each iteration.

#### 3.3.3. Selection

The performance of the algorithm much depends on the successful selection operation of ECPs. In this stage, nECPI number of particles are arbitrarily selected from the generated population, sorted according to their fitness, starting from the best to the worst. The particles selected in this phase will go through the next phase- interaction.

#### 3.3.4. Interaction

As aforementioned, not all ECPs interact, only few of them do which are specified by nECPI. In the interaction phase, the selected nECPI particles interact among themselves, in one of the predetermined strategies. To illustrate clearly, let us assume that nECPI = 3 which are sorted as best to worst and denoted as ‘ECP_1′_, ‘ECP_2′_, and ‘ECP_3′_. The best particle among all the particles is denoted as ECP_best_. It is worth mentioning that the procedure will remain same for any other value of nECPI. Although any value for nECPI can be used, from the authors experience nECPI = 2 and nECPI = 3 give good results compared to other values.

##### Strategy 1

**Figure 1:**
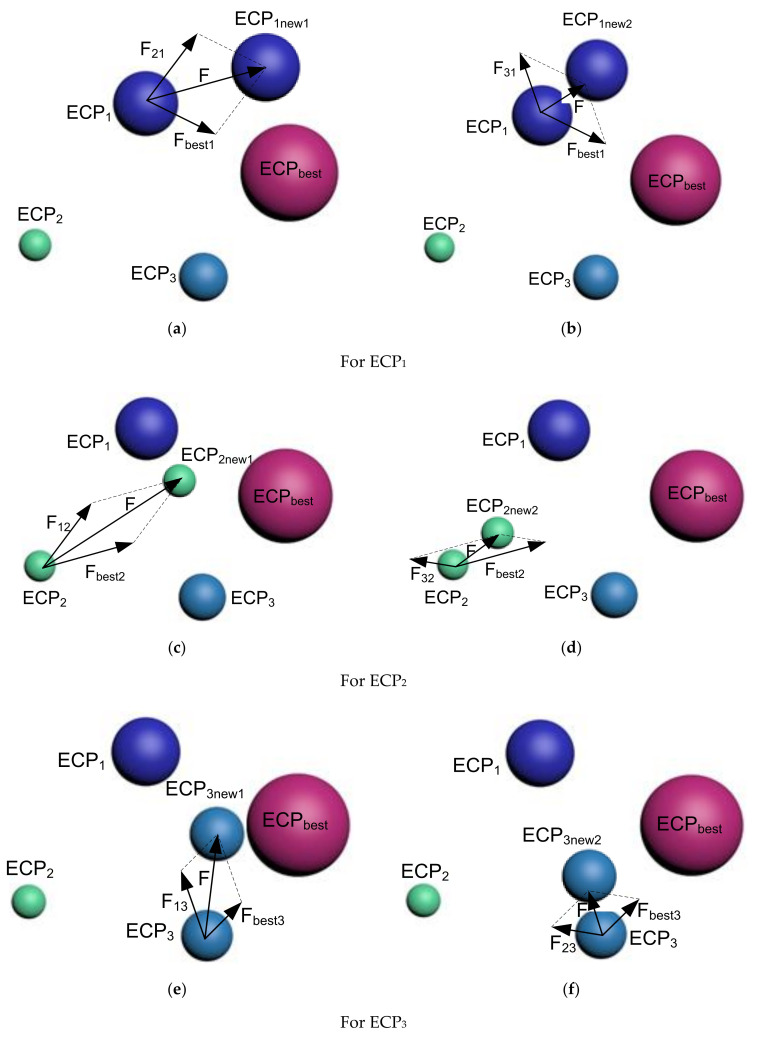
Illustration of the interaction between ECPs for strategy 1.

In strategy 1, only the best ECP, also known as the ${\mathrm{ECP}}_{\mathrm{best}}$ gets interacted with another ECP at the time. Therefore, this strategy generates two new ECPs (${\mathrm{ECP}}_{i\mathrm{new}}{}_{1}$ and ${\mathrm{ECP}}_{i\mathrm{new}}{}_{2})\;$for the case of three interacting ECPs. The whole procedure is presented in Figure 1.

For ${\mathrm{ECP}}_{1}$:

At first, it gets affected by ECP_2_ and ECP_best,_ simultaneously, to move to ${\mathrm{ECP}}_{1\mathrm{new}}{}_{1}$. Then, ECP_3_ and ECP_best_ affected ECP_1_ to move to ${\mathrm{ECP}}_{1\mathrm{new}}{}_{2}$.

The required force to move ECP_1_ up to ${\mathrm{ECP}}_{1\mathrm{new}}{}_{1}$ is represented as:

(10) $$F=F_{best1}+F_{21}$$

where: $F_{21}$ represents the force of ECP_2_ on charge particle ECP_1_ whereas $F_{\mathrm{best}1}$ is the force on ECP_1_ from ECP_best_.

These two forces can be expressed as follows:

(11) $$F_{best1}=\beta \times \left(ECP_{best}-ECP_{1}\right)$$

(12) $$F_{21}=\beta \times \left(ECP_{1}-ECP_{2}\right)$$

where β is a randomly generated number through any of the distribution mechanisms, such as normal distribution or gamma distribution.

As discussed earlier, ECP_best_ attracts ECP_1_, whereas ECP_2_ repeals ECP_1_, because ECP_1_ is better than ECP_2_ and worse than ECP_best_.

Therefore, the total force pushing ECP_1_ towards ${\mathrm{ECP}}_{1\mathrm{new}}{}_{1}$ is given by (as demonstrated in Figure 1a):

(13) $$\begin{array}{cc} ECP_{1new}{}_{1} & =ECP_{1}+F\\ & =ECP_{1}+F_{best1}+F_{21}\\ & =ECP_{1}+\beta \times \left(ECP_{best}-ECP_{1}\right)+\beta \times \left(ECP_{1}-ECP_{2}\right) \end{array}$$

Similarly, the required force pushing ECP_1_ towards ${\mathrm{ECP}}_{1\mathrm{new}}{}_{2}$ is as follows:

(14) $$\begin{array}{cc} ECP_{1new}{}_{2} & =ECP_{1}+F\\ & =ECP_{1}+F_{best1}+F_{31}\\ & =ECP_{1}+\beta \times \left(ECP_{best}-ECP_{1}\right)+\beta \times \left(ECP_{1}-ECP_{3}\right) \end{array}$$

It is illustrated in Figure 1b.

For ${\mathrm{ECP}}_{2}\;$:

ECP_1_ and ECP_best_ affects simultaneously to move to ${\mathrm{ECP}}_{2\mathrm{new}}{}_{1}$. Similarly, ECP_2_ is affected by ECP_3_ and ECP_best_ simultaneously for moving to ${\mathrm{ECP}}_{2\mathrm{new}}{}_{1}$. These two scenarios are illustrated in Figure 1c,d, respectively.

For ${\mathrm{ECP}}_{3}\;$:

This last and third particle, ECP_3_ is affected by ECP_1_ and ECP_best_, simultaneously, to move to the new particle, ${\mathrm{ECP}}_{3\mathrm{new}}{}_{1}$. Later, ECP_3_ gets affected by ECP_2_ and ECP_best_ together to create ${\mathrm{ECP}}_{3\mathrm{new}}{}_{2}$ (Figure 1e,f).

Equations (13) and (14), used for ECP1, are like the ones used for ECP_2_ and ECP_3_. They have not been rewritten here to avoid repetition.

##### Strategy 2

In the second strategy, the ECP_best_ does not interact with all of the remaining ECPs; rather, it associates with selected ones. Therefore, in the presented illustration, all the three interacting ECPs will create one new ECP, each, which is called ${\mathrm{ECP}}_{i\mathrm{new}}$ (where i represents the indexing of the selected ECP).

For ${\mathrm{ECP}}_{1}$:

ECP_1_ gets affected by ECP_2_ and ECP_3,_ simultaneously, which will lead it to move towards ${\mathrm{ECP}}_{1\mathrm{new}}$. The resulting force for this movement is given by:

(15) $$F=F_{21}+F_{31}$$

where: $F_{21}$ refers to the force of ECP_2_ for ECP_1_ and $F_{31}$ represents the force of ECP_3_ on the first charged particle, ECP_1_.

**Figure 2:**
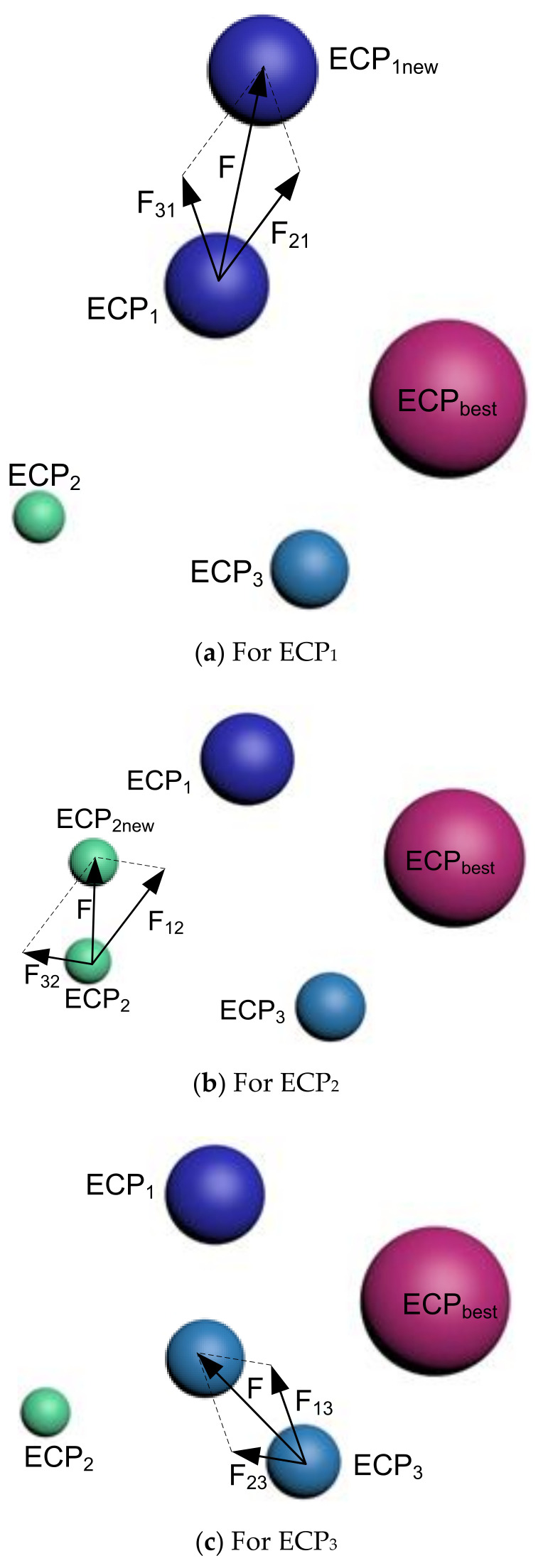
Interaction between ECPs—(**a**) ECP_1_, (**b**) ECP_2_, and (**c**) ECP_3_ for Strategy 2.

Therefore, the required force of pushing ECP_1_ towards ${\mathrm{ECP}}_{1\mathrm{new}}$ is given by (as shown in Figure 2a):

(16) $$\begin{array}{cc} ECP_{1new} & =ECP_{1}+F_{1}\\ & =ECP_{1}+F_{21}+F_{31}\\ & =ECP_{1}+\beta \times \left(ECP_{1}-ECP_{2}\right)+\beta \times \left(ECP_{1}-ECP_{3}\right) \end{array}$$

For ${\mathrm{ECP}}_{2}$:

The second particle (ECP_2_) is affected by ECP_1_ and ECP_3,_ simultaneously, to create the movement towards ${\mathrm{ECP}}_{2\mathrm{new}}$. The resulting force for this incident is denoted by:

(17) $$F=F_{12}+F_{32}$$

where: $F_{12}$ refers to the force between ECP_1_ and ECP_2_ whereas $F_{32}$ represents the force between ECP_3_ and ECP_2_.

For ${\mathrm{ECP}}_{3}$:

The ECP3 gets affected by first and second particles, together, with the following force:

(18) $$F=F_{13}+F_{23}$$

where: $F_{13}$ is the force between ECP_1_ and ECP_3_ whereas $F_{23}$ represents the force between ECP_2_ and ECP_3_.

Figure 2b,c represents the forces to move ECP_2_ to ECP_2new_ and ECP_3_ to ECP_3new_, respectively. At the same time, the equation of the force equation will be like the one presented in (16).

##### Strategy 3

This strategy uses Strategies 1 and 2, both in generating the new ECPs. Therefore, in the case of nECPI = 3, a total of 9 new ECPs will be available, where strategies 1 and 2 will generate 6 and 3 ECPs, respectively.

The product of the interaction phase is a new population of ECPs, which is denoted as newECP. Its size is the same as the original population size, whatever the nECPI or the interaction strategy. This means that the product of the interaction phase will generate, for instance, 35 particles if the initially generated population is 35 particles.

#### 3.3.5. Checking the Bounds

This phase checks whether any new ECPs created in the interaction phase have been generated outside the search space. To verify this, all of the new ECPs are checked. If any particles are found outside the search space, they will be brought back within the assigned boundary.

#### 3.3.6. Diversification

Some part of the new ECP population needs to be diversified with a specific probability, known as the probability of diversification (Pd). In this algorithm, the diversity operator collects information from newECP and archiveECP. The pseudocode of the diversification phase is presented in Algorithm 2.

#### 3.3.7. Diversification

Some part of the new ECP population needs to be diversified with a specific probability, which is known as probability of diversification (Pd). In this algorithm, the diversity operator collects information from newECP and archiveECP. The pseudo code of the diversification phase is presented below in Algorithm 2.
**Algorithm 2** Pseudocode for the diversification phase1 **For**i=1: newECP 2     **For**j=1: ProblemSize
3        If rand<Pd
4            select a random ECP from the archive pool (k)
5           $\mathrm{newECP}\left(i,j\right)=\;\mathrm{archiveECP}\left(k,j\right)$ 6        **End If** 7 **End For**

#### 3.3.8. Population Update

The new population is modified in this phase and stored in the previously created archive pool. The best nECP particles, from rank 1 to nECO, will generate the new population. It will go through an iterative procedure again, as described before.

#### 3.3.9. Criteria for Termination

There could be several ways of terminating for the discussed algorithm. However, in this current ECPO version, the iteration continues for a specified number of times (MaxITER) and then stops. The MaxITER is an important parameter for the ECPO-ME algorithm. It is determined by trying various values and selecting the optimal one. It is obvious that increasing MaxITER will improve the results in most cases, but, at the same time, it will take more time to get the results. Inversely, decreasing MaxITER might lead to less efficient results, since the algorithm might need more iteration to converge to the global minimum. It is therefore suggested to optimally determine the MaxITER parameter.

#### 3.3.10. Constraint Handling

The constraints in ECPO are handled by changing the constrained optimization problems to unconstrained using the penalty factor technique. In this technique, if an ECP fails to fulfill the constraint demand, a penalty factor will be imposed on the objective function. The higher the severity of the violation, the bigger will be the penalty term.

#### 3.3.11. Motion Encoding (ME)

ECPO has yet to undergo any modification or upgrade as a new metaheuristic optimization algorithm. However, this paper has added a motion-encoding feature to the existing approach [26] to improve the search capability of ECPO. It has helped the algorithm cope with the challenging task of searching the dynamic targets within a limited time frame. Therefore, there is the need to encode the particle positions to find the global solution easily. To achieve this, a position needs to be defined as a multi-dimensional vector that can represent the search path [24].

In this paper, UAV motion is used to encode the particle positions. Each searching path is thus seen as a set of UAV motion segments instead of the traditional nodal concept. Thus, the movement of a UAV from one cell to another is tracked accordingly to run the optimization process. The whole mapping procedure of ECPO-ME is such that the particles search the motion space, not the cartesian space.

## 4. Application, Results, and Discussion

In this section, the performance of the proposed ECPO-ME-based search algorithm is assessed under different scenarios and compared with several other optimization algorithms. All the scenarios and algorithms have been implemented and simulated using the commercial software MATLAB. All calculations were done using a computer equipped with Inter Core i7- 6500U CPU, 2.59 GHz, 8 GB RAM, running the Windows 10 operating system.

### 4.1. Scenarios

**Figure 3:**
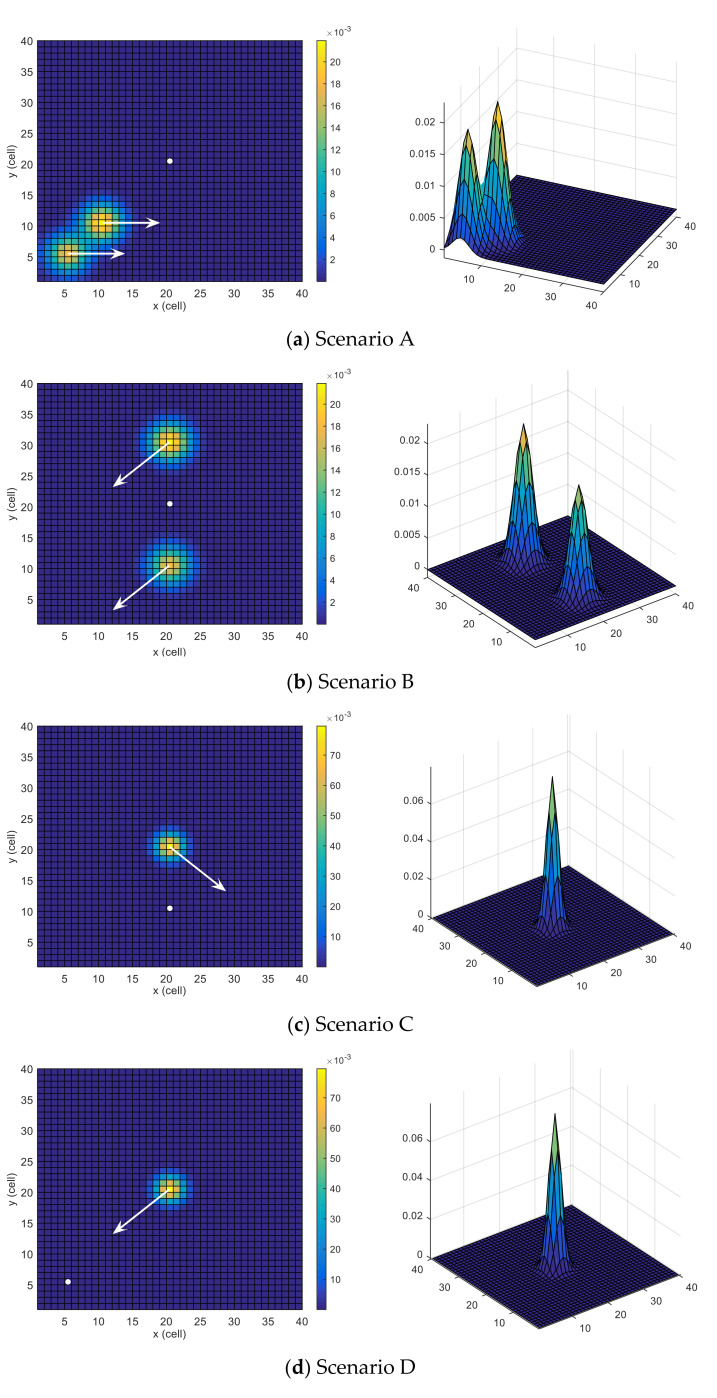

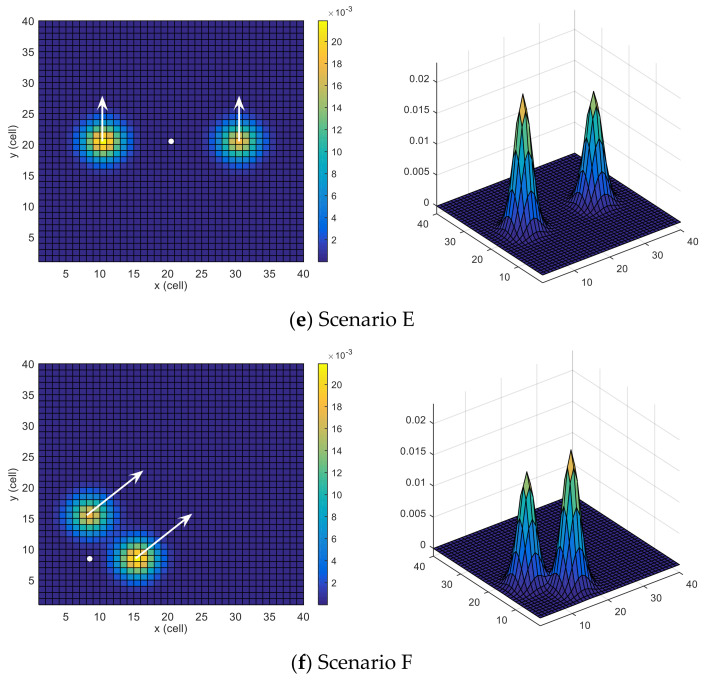
Search scenarios.

Six scenarios are used, some of which are similar to the ones investigated in [24]. All the scenarios have same grid size $\left(w_{x}=w_{y}=40\right)$ but different initial position (or location) of the UAV, target motion model $P(x_{t}|x_{t-1})\;$and belief map $b\left(x_{0}\right)$. The tested scenarios are depicted in Figure 3. The probability map is color-coded (cells with higher probabilities of target presence are colored with warmer colors), the UAV’s initial position is indicated with a white circle, and the dynamics of the moving target are outlined with white arrows.

**Scenario A:** This scenario has two areas with higher probabilities, located near each other and moving towards the east. However, the likelihood of the upper area is slightly higher than the lower area. The UAV is situated in the middle of the search space. This scenario is depicted in Figure 3a.

**Scenario B:** In this scenario, there are two areas with high probabilities that are equally spaced from the initial position of the UAV along the *y*-axis of the search space. However, the probability of the upper area is slightly higher than that of the lower area. Both areas move towards the south-west. The UAV is located at the middle of the search space, and the corresponding scenario is depicted in Figure 3b.

**Scenario C:** In this scenario, picturized in Figure 3c, only one dense area moves towards the southeast. This scenario tests the adaptability of the searching algorithm. The UAV is located at the middle of the *x*-axis and under the target along the *y*-axis of the search space.

**Scenario D:** In this scenario, there is only one dense area, like in Scenario C. However, the target is moving towards the UAV in this scenario, which is initially located at the south-west region of the search space. This scenario is depicted in Figure 3d.

**Scenario E:** This scenario, depicted in Figure 3e, consists of two probability areas that are equally spaced from the initial position of the UAV along the *x*-axis of the search space, and the target is moving towards the north. However, the probability of the right area is slightly higher than that of the left area. The UAV is located in the middle of the search space.

**Scenario F:** In this scenario, two probability areas are the same distance apart from the initial position of the UAV, and the target is moving towards the north-east. However, the probability of the lower area is slightly higher than the upper area. The UAV, along with the areas, is on the south-west side of the search space. This scenario is depicted in Figure 3f.

### 4.2. Comparing Algorithms

As mentioned before, the proposed algorithm results are compared to ten well-known algorithms, which are described in the following subsections.

Motion-encoded particle swarm optimization (MPSO): This algorithm is an improved version of the famous PSO algorithm using the ME mechanism. PSO is inspired by the social behavior of bird flocking or fish schooling [27,28]. In the PSO a population of particles (each particle has a position and a velocity) evolves during iterations. During each iteration, the position and velocity of each particle are updated using the best solution achieved by each particle so far, and the overall best solution. The MPSO has been used in [24] to solve a similar problem to the one solved in this paper, i.e., finding a moving target using UAVs. As reported in [24] the obtained results using MPSO were better than some versions of the PSO and many other optimization algorithms. Therefore, it will be interesting to compare the results obtained using the proposed approach with those obtained using MPSO.

Most-valuable-player algorithm (MVPA): This algorithm is an optimization algorithm that optimizes the problem at hand by considering a population of players that compete against each other (individually and in teams) to win the trophy of the most valuable player (individually) and the championship (in teams) [29].

Differential evolution (DE): DE is a population-based optimization algorithm relying on a variety of evolutionary processes. A specific member of the population, representing the solution, iteratively improves itself from one generation to another. This algorithm is famous for its stability and versatility, specifically among population-based search algorithms [30]. The standard DE method does not require gradient information trivializing the condition of differentiability. After the occurrence of a randomly chosen evolutionary event, if a candidate gives a better solution, the adaptation is accepted for that candidate; otherwise, its traits remain unchanged for the next generation. The process is repeated until pre-defined termination criteria are met.

Genetic algorithm (GA): GA was first used by Holland in 1975 based on Darwin’s theory of evolution [31]. Now it is one of the most famous metaheuristics and has been used in many research fields. The objective of the GA is to maximize the payoff of candidate solutions in the population against a cost function (or a fitness function) from the problem domain using three operators: selection, recombination, and mutation.

Electrostatic discharge algorithm (ESDA): This algorithm is an optimization algorithm inspired by electrostatic discharge (ESD). In this algorithm, objects (population of objects) work in a given environment (search space). If these objects are near each other, the ESD may occur (sometimes between two objects and sometimes between three objects). If ESD occurs on a victim object couple of times, the object or some of its components is/are damaged, and it/they must be changed [32].

Biogeography-based optimization (BBO): BBO is also an evolutionary algorithm inspired by nature. As evident from the name, its equations are modeled on biogeography, which covers the distribution and growth of living beings through space and time. Three events decide the fitness of each candidate in every generation: speciation, migration, and extinction. Like DE, BBO maintains the population of candidates solutions after every biogeographic event [33]. Once the termination criterion is met, the candidate with the best objective function is considered the optimal solution.

Artificial bee colony (ABC): ABC is another nature-inspired optimization algorithm based on the foraging pattern of a bee swarm. The bees in a colony belong to either of the three groups; employed, onlookers, and scouts. The employed ones look for the food source around the hive, where the location of the food represents the optimal solution, and the amount of nectar in a food source is equivalent to the fitness value. Once all employed bees bring the food, the onlookers evaluate the quantity of food and determine the next trip of employed bees. Scouts replace a depleted source with a randomly located new food source [34]. All these metaphors are implemented mathematically and iteratively give the optimal solution in a search space.

Gravitational search algorithm (GSA): GSA models the optimization formulation using Newton’s gravitational laws and laws of motion. Many variants of GSA have been obtained by modifying its parameters, including Kbest, velocity, and position. The mass interaction under Newtonian mechanics governs the convergence of the solution. Every mass exerts a force on another mass under a fixed gravity determining the fitness function. Based on the interaction, the velocity and position of each agent are updated [35]. The process continues till the termination criteria are met. Ultimately, the solution converges to the optimal solution.

Teaching–learning-based optimization (TLBO): The TLBO algorithm is inspired by the teaching-learning process. In this algorithm, there are two phases, namely the teacher phase and the learner phase. In the first phase, the population of students improves their knowledge with the teacher who is the best solution among the population. However, in the learner phase, the students interact to improve their knowledge [36].

Black-hole (BH) algorithm: This algorithm is inspired by the black hole theory, a region of spacetime where no object, particle, or even light can escape from it due to its strong gravity. In this algorithm, the population of stars moves in space. If a star approaches the black hole (within a radius called the Schwarzschild radius), it is absorbed by the hole, and a new star is generated to keep the same number of stars over the iterations [37,38].

### 4.3. Results

The proposed algorithm and the comparing ones were applied to the UAV dynamic target search. Each algorithm has been run 30 times with a population of size 30 and 300 as the maximum number of iterations. The remaining parameters related to each algorithm are selected as recommended in the literature. Table 1 presents the ‘BEST’, the ‘MEAN’, the ‘MEDIAN, the ‘WORST’, the ‘Standard Deviation (SD)’ and the ‘Feasibility Ratio (FR)’ for each algorithm and each scenario. The FR is defined as the percentage of the successful runs over the total number of attempted runs. The following comments can be made from Table 1:

- For Scenario A, the proposed ECPO-ME algorithm obtained the best results for the ‘BEST’, the ‘MEAN’ and, the ‘MEDIAN values. The TLBO algorithm obtained the highest ‘WORST’ and ‘SD’ Values.
- For Scenario B, the TLBO obtained a slightly better value than the ECPO-ME in terms of the ‘BEST’ values. However, the ECPO-ME obtained the best results of the ‘MEAN’, the ‘MEDIAN, the ‘WORST’ and the ‘SD’ values compared to the remaining algorithms.
- For Scenario C and Scenario D, the proposed ECPO-ME outperformed the other algorithms in terms of the statistical performance indicators used in this study. It is worth mentioning that the DE achieved equally good results to ECPO-ME in terms of the ‘BEST’ values.
- For Scenario E, the proposed ECPO-ME showed better performance than all the remaining algorithms in terms of the ‘BEST’, the ‘MEAN’ and the ‘MEDIAN values while the DE achieved better results in terms of the ‘WORST’ and the ‘SD’ values.
- For Scenario F, the proposed ECPO-ME achieved a better result than all the remaining algorithms in terms of the ‘BEST’, the ‘MEDIAN’ and the ‘WORST’ values, while the TLBO was better in terms of the ‘MEAN’ and ‘SD’ values.
- All algorithms gave an FR equal to 100, which reflects that all of them could find a solution (i.e., a path) in all the runs and for all the investigated scenarios except for the ABC algorithm for Scenario D (FR = 93.33%).
- For scenarios with high probability regions, like Scenario C and Scenario D, the likelihood of finding the target is higher because there is no need to divide or spread the chances of finding the target in other areas.

**Figure 4:**
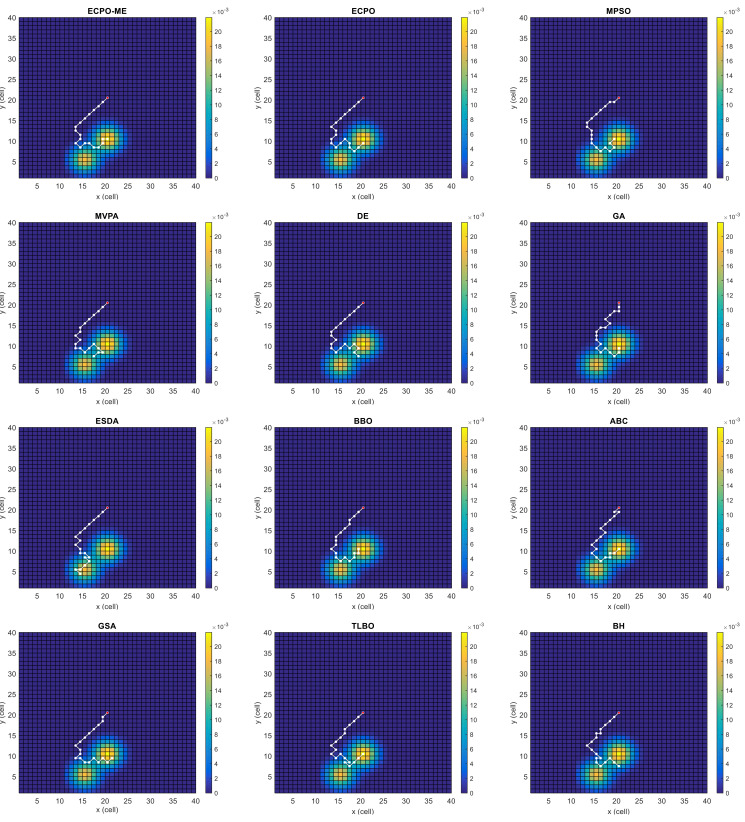
Search paths for Scenario A, obtained using the tested algorithms.

**Figure 5:**
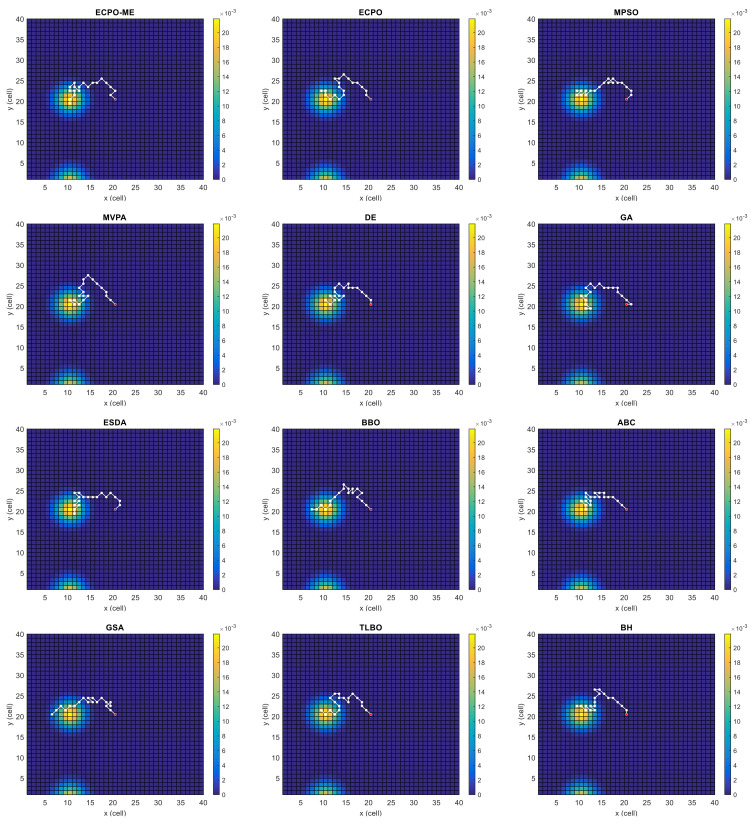
Search paths for Scenario B, obtained using the tested algorithms.

**Figure 6:**
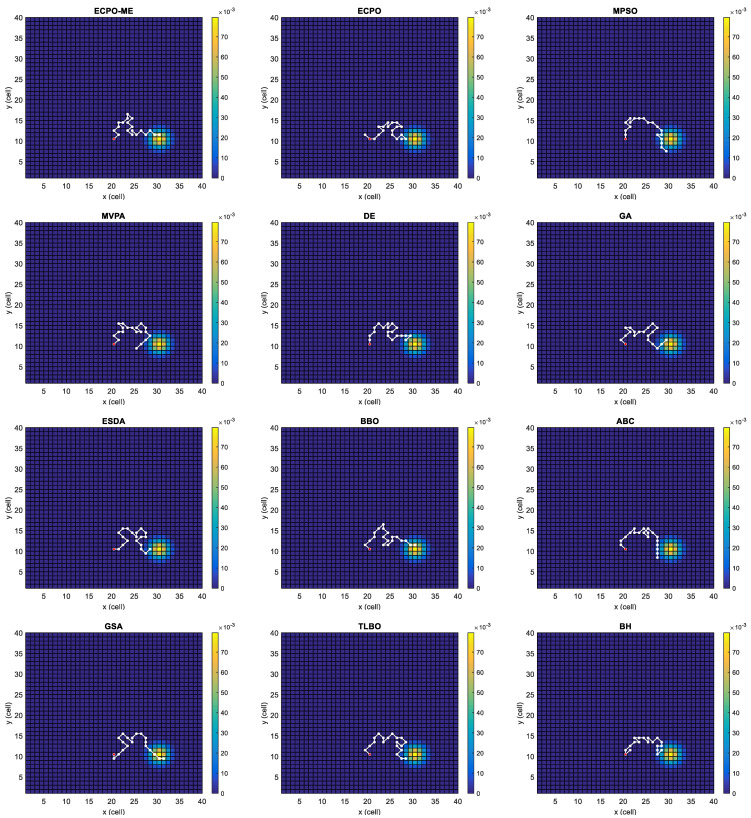
Search paths for Scenario C, obtained using the tested algorithms.

**Figure 7:**
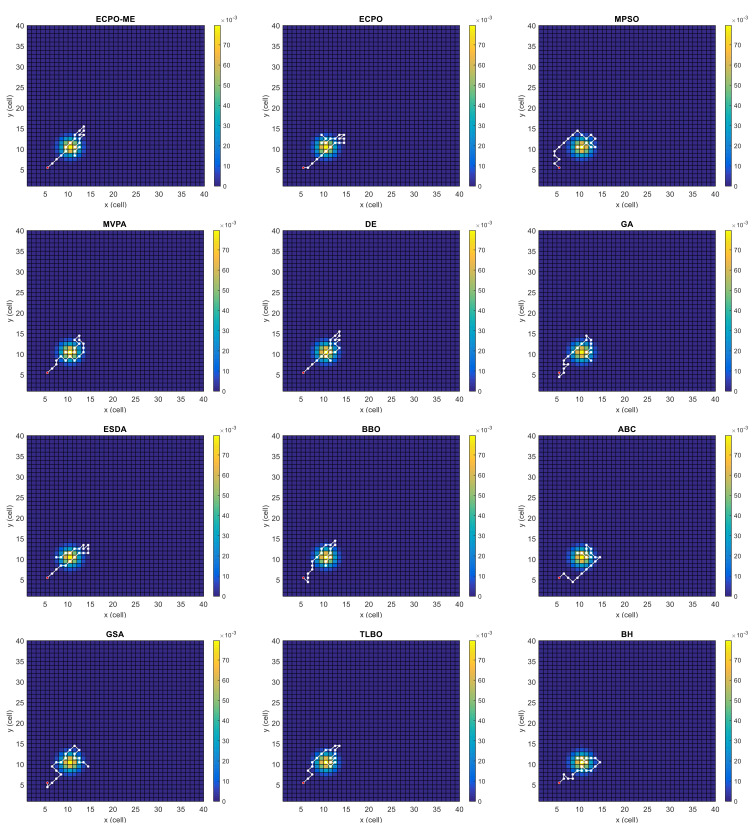
Search paths for Scenario D, obtained using the tested algorithms.

**Figure 8:**
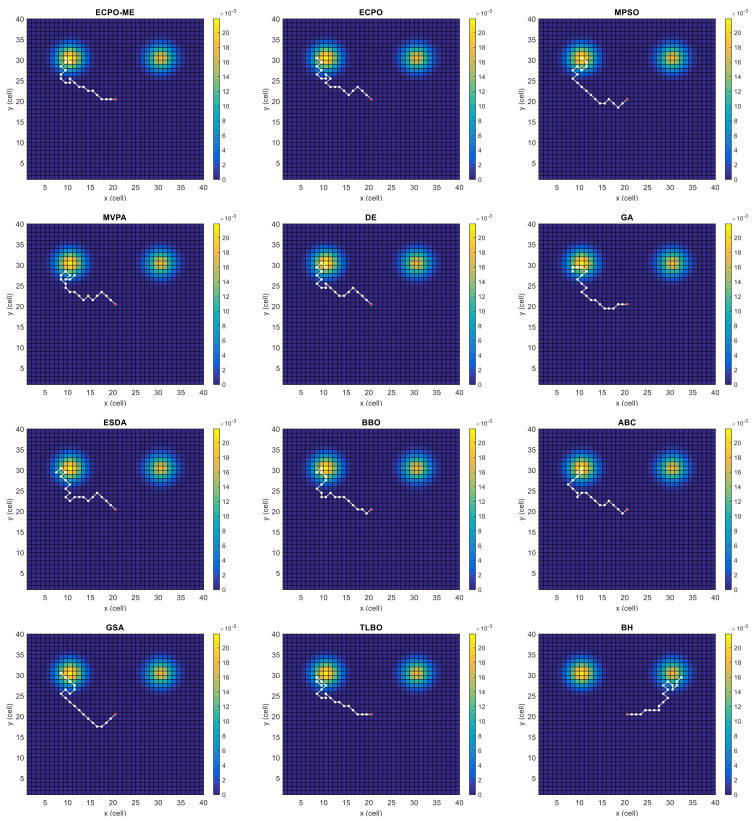
Search paths for Scenario E, obtained using the tested algorithms.

**Figure 9:**
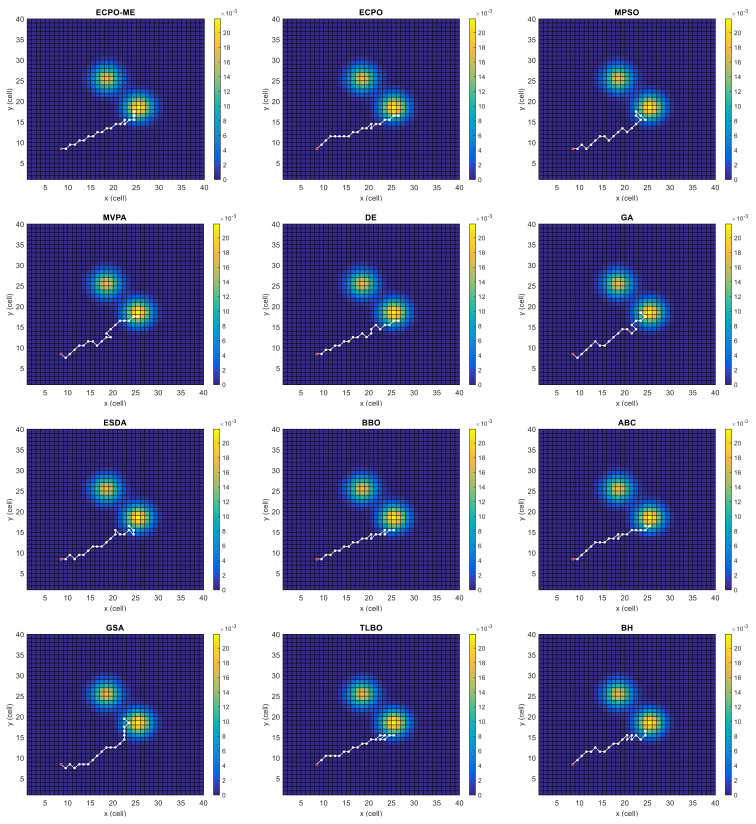
Search paths for Scenario F, obtained using the tested algorithms.

Figure 4, Figure 5, Figure 6, Figure 7, Figure 8 and Figure 9 depict the optimal search paths obtained for the tested algorithms for Scenario A, Scenario B, Scenario C, Scenario D, Scenario E, and Scenario F, respectively. In these figures, the sketched path represents the searching path. However, the belief map reflects the target at the last step. This map can be compared with the initial map, illustrated in Figure 3, to see the evolution of the target. It can be seen from these figures that, in all scenarios, the proposed ECPO-ME algorithm was capable of following the target paths and, thus, finding out the highest probability region.

**Figure 10:**
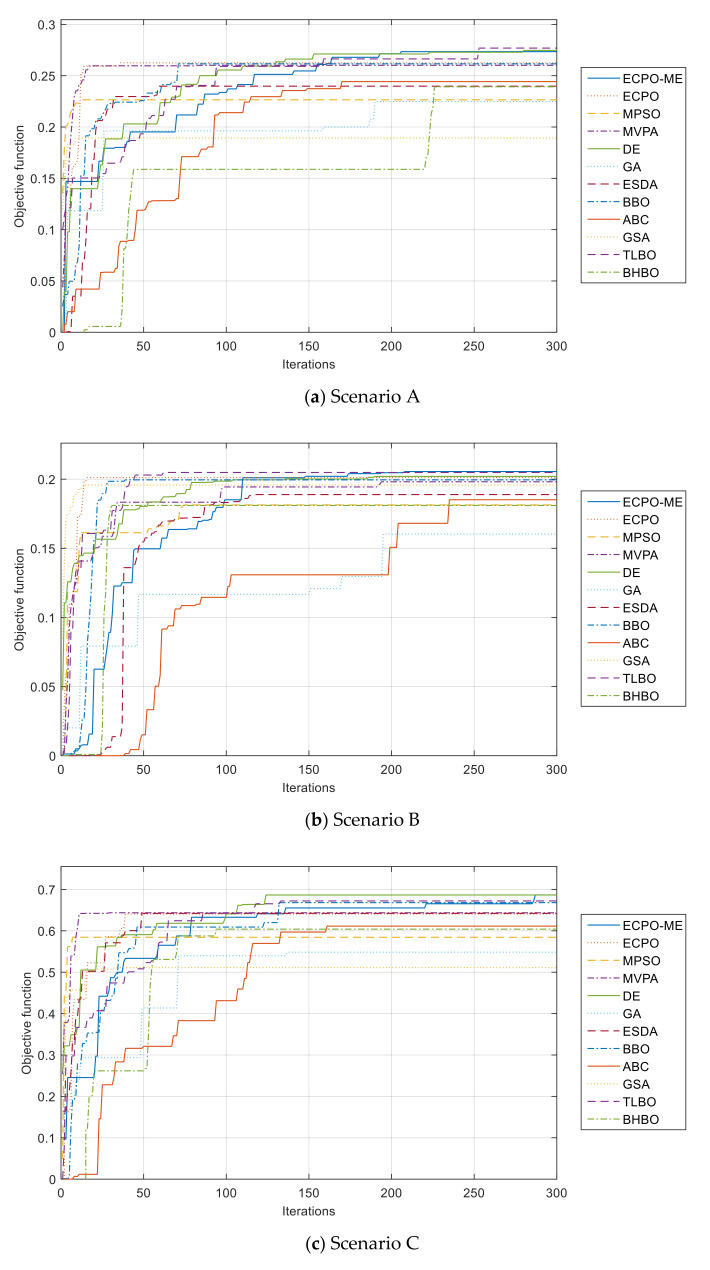

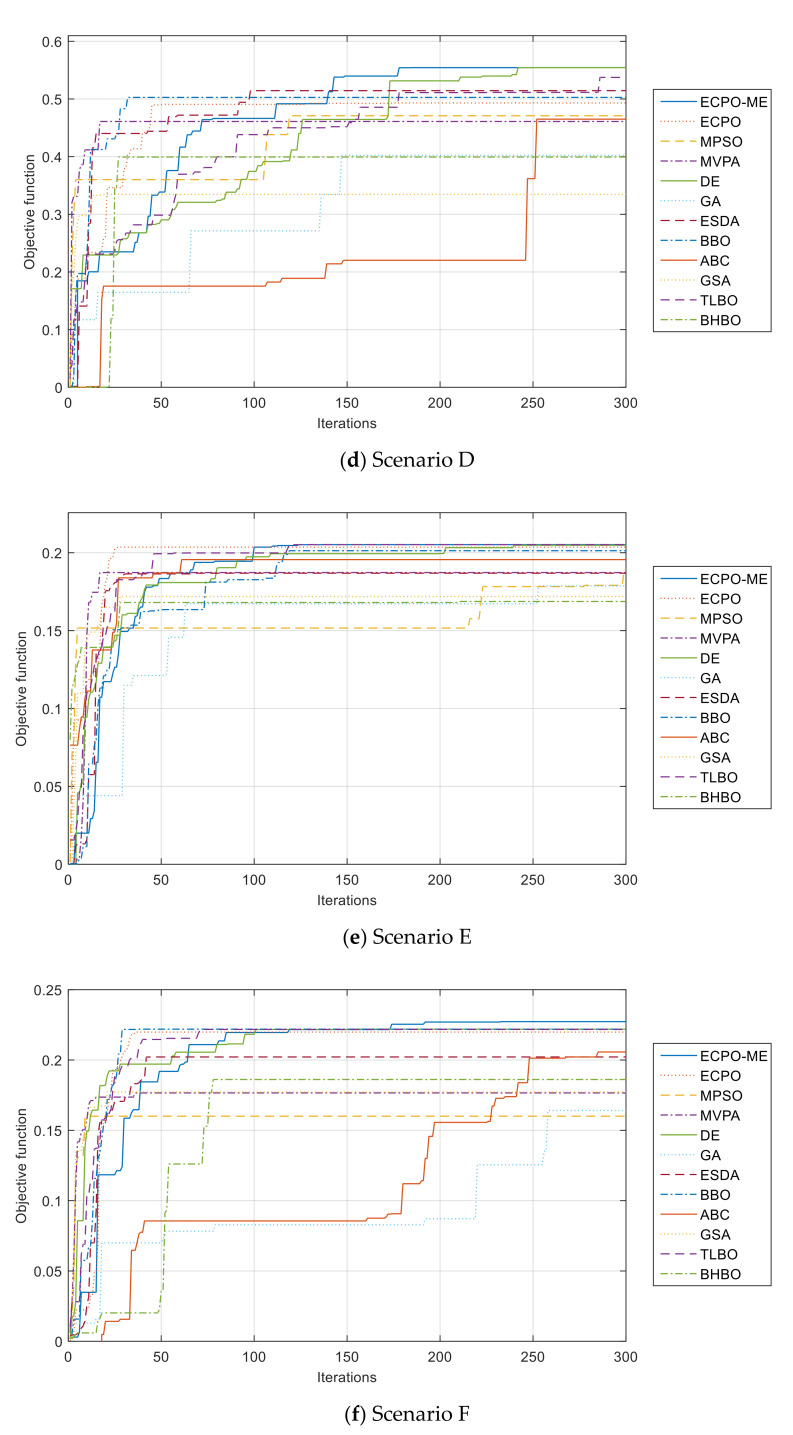
Convergence curves of the ECPO-ME for all scenarios.

Furthermore, the convergence curves of all algorithms are given in Figure 10. It can be seen from these curves that the ECPO-ME algorithm achieves the best results in almost all the investigated scenarios.

### 4.4. Discussion

The analysis of the obtained results leads to the conclusion that the proposed ECPO-ME algorithm has capability to perform better than many of the well-known and widely used optimization algorithms. For instance, for Scenario A, the ECPO-ME has outperformed the remaining algorithms by 13.30%, 3.74%, 1.75%, 28.20%, 8.82%, 3.02%, 11.02%, 5.00%, 0.32%, 13.54%, respectively. Another example is for Scenario B, the ECPO-ME has outperformed the remaining algorithms (except TLBO) by 5.26%, 21.99%, 6.23%, 0.61%, 22.89%, 15.25%, 5.65%, 13.14%, 46.02%, and 15.53%, respectively.

The success of the ECPO-ME lies first in the search ability of the ECPO itself and secondly, in the motion-encoding mechanism introduced to the ECPO for the first time.

It has been proven earlier that ECPO is superior in terms of its ability to search for the optimal solution, and hence ranked among the top five algorithms in doing so. Yet, the proposed enhancement, ECPO-ME outperforms its predecessor in terms of its search ability, one of the primary reasons for ECPO-ME’s superior performance, as recorded in Table 1.

For the second point, implementing the motion-encoding mechanism with the ECPO has improved its performance for finding a moving target with a UAV. The ME mechanism has prevented the algorithm from generating invalid paths during the search process. Furthermore, the ME mechanism has transformed the search procedure from a cartesian space to a motion space, which has improved the adaptation of ECPO-ME to the dynamics of the target.

## 5. Conclusions

In this work, a new approach based on the electric charged particles optimization algorithm and the motion-encoding mechanism is presented to search the moving targets using unmanned aerial vehicles. The ME mechanism transforms the cartesian problem into a motion-based problem. The search path is then transformed into a series of motions where the UAV is restricted to the neighbor cells of the current location cell. Extensive simulations (six different scenarios with different complexities) and comprehensive comparisons with other well-known optimization algorithms (ten algorithms) have shown that the proposed approach is highly effective and reliable in searching moving targets using UAVs. Such an approach is highly suitable to use in cattle management, where the animals can be considered the moving targets that are prone to get lost while grazing.

The challenges for future works are multiple. One axis is to investigate the use of multiple UAVs instead of a single UAV, as done in this paper. Many approaches can be adopted, like allocating each UAV to a region, or having all of them search the highest regions. Another interesting point can be the investigation of information sharing between the UAVs, which may significantly improve the search procedure. Another challenging task or research axis can be extending this study to multiple moving targets instead of just one or two targets. The mixture of both axis of research, i.e., using multiple UAVs to search for multiple targets, can lead to a large-scale optimization problem, which is another challenging task to be investigated in the future.

Furthermore, in this paper, only one objective has been considered, with the cumulative probability, $P_{t}$. Including other objectives, such as fuel consumption by the UAVs or avoidance of any forbidden areas, can define future work extending that reported here. The problem in hand will then become a multi-objective one. From an application point-of-view, this research can be extended to a real-life scenario, wherein the motion of a moving animal can be detected to communicate its location to a nearby control center for further action.

## Figures and Tables

**Table 1:** Statistical analysis of the obtained results using each algorithm. The bold in table shows the best results.

		ECPO-ME	ECPO	MPSO	MVPA	DE	GA	ESDA	BBO	ABC	GSA	TLBO	BH
Scenario A	BEST	**0.20551**	0.20116	0.18138	0.19810	0.20197	0.16030	0.18885	0.19948	0.18511	0.19572	0.20485	0.18101
	MEAN	**0.19239**	0.16555	0.06021	0.11085	0.18960	0.13368	0.14730	0.17851	0.14966	0.04258	0.19207	0.13570
	MEDIAN	**0.19118**	0.18091	0.03225	0.12225	0.18942	0.13315	0.15159	0.18132	0.16003	0.00095	0.19115	0.13742
	WORST	0.18209	0.03135	0.00000	0.00002	0.17250	0.10551	0.09942	0.11788	0.00131	0.00000	**0.18303**	0.06931
	SD	0.00593	0.04174	0.06305	0.06100	0.00630	0.01466	0.02609	0.01696	0.03707	0.06203	**0.00587**	0.02547
	FR	100	100	100	100	100	100	100	100	100	100	100	100
Scenario B	BEST	0.27634	0.26252	0.22652	0.26014	0.27466	0.22486	0.23978	0.26156	0.24424	0.18925	**0.27689**	0.23919
	MEAN	**0.25724**	0.23126	0.10670	0.17272	0.24516	0.18563	0.18328	0.21522	0.19452	0.05717	0.25216	0.19299
	MEDIAN	**0.25820**	0.24153	0.11274	0.19613	0.24909	0.18932	0.18379	0.23196	0.20196	0.04139	0.25462	0.19735
	WORST	**0.23530**	0.14698	0.01105	0.03688	0.14928	0.15175	0.11802	0.11055	0.01471	0.00009	0.22876	0.13430
	SD	**0.00964**	0.02539	0.05659	0.06069	0.02339	0.01860	0.03786	0.04466	0.04892	0.05281	0.01293	0.02803
	FR	100	100	100	100	100	100	100	100	100	100	100	100
Scenario C	BEST	**0.68662**	0.64070	0.58442	0.64361	**0.68662**	0.54835	0.64221	0.66811	0.61142	0.51114	0.67221	0.60402
	MEAN	**0.64158**	0.52614	0.30143	0.37015	0.62170	0.46797	0.47561	0.55109	0.49444	0.22860	0.63269	0.49182
	MEDIAN	**0.64997**	0.55979	0.31420	0.39419	0.63538	0.47210	0.48670	0.59358	0.52423	0.23693	0.63631	0.50913
	WORST	**0.57162**	0.26147	0.00240	0.01434	0.35432	0.36236	0.27549	0.23189	0.26933	0.00000	0.55967	0.36322
	SD	**0.02876**	0.10643	0.17289	0.15426	0.06193	0.05306	0.08845	0.10633	0.09406	0.13620	0.03111	0.07498
	FR	100	100	100	100	100	100	100	100	100	100	100	100
Scenario D	BEST	**0.55431**	0.49309	0.47090	0.46095	**0.55431**	0.40220	0.51444	0.50274	0.46497	0.33458	0.53742	0.39930
	MEAN	**0.48849**	0.38766	0.24390	0.31673	0.45452	0.29675	0.31233	0.35144	-	0.20418	0.46575	0.28810
	MEDIAN	**0.49887**	0.38931	0.23998	0.32675	0.44347	0.29698	0.30451	0.33692	-	0.20486	0.46263	0.27994
	WORST	**0.40148**	0.26634	0.04982	0.09241	0.32458	0.21807	0.18826	0.22614	-	0.00002	0.37301	0.18647
	SD	**0.03120**	0.05620	0.08177	0.09298	0.06339	0.04411	0.07879	0.07564	-	0.08891	0.04044	0.05650
	FR	100	100	100	100	100	100	100	100	93.33	100	100	100
Scenario E	BEST	**0.20518**	0.20358	0.18901	0.18726	0.20477	0.17868	0.18686	0.20130	0.19559	0.17188	0.20518	0.16870
	MEAN	**0.19008**	0.17367	0.11979	0.13986	0.18615	0.13753	0.14400	0.17809	0.16200	0.07077	0.18893	0.13598
	MEDIAN	**0.18870**	0.18154	0.12904	0.14625	0.18395	0.14029	0.14865	0.17725	0.16468	0.07634	0.18830	0.13544
	WORST	0.17187	0.11000	0.00021	0.02299	**0.17277**	0.09305	0.04521	0.15401	0.04478	0.00012	0.16969	0.07844
	SD	0.00801	0.02362	0.05092	0.03505	**0.00787**	0.01839	0.03434	0.01107	0.02810	0.05553	0.00867	0.02228
	FR	100	100	100	100	100	100	100	100	100	100	100	100
Scenario F	BEST	**0.22728**	0.21985	0.16005	0.17661	0.22195	0.16416	0.20217	0.22195	0.20572	0.17721	0.22175	0.18615
	MEAN	0.20007	0.18020	0.07334	0.10851	0.19650	0.10638	0.13038	0.19142	0.15022	0.06385	**0.20008**	0.13070
	MEDIAN	**0.21024**	0.18573	0.07296	0.13480	0.20160	0.11310	0.14440	0.20465	0.15892	0.03687	0.20930	0.13941
	WORST	**0.16650**	0.08188	0.00129	0.00564	0.13640	0.04128	0.03171	0.08111	0.00041	0.00070	0.16648	0.02830
	SD	0.01978	0.03226	0.05025	0.05427	0.02234	0.03378	0.04813	0.03334	0.05207	0.06128	**0.01820**	0.04135
	FR	100	100	100	100	100	100	100	100	100	100	100	100
